# Editorial: Digital interventions and serious mobile games for health in low- and middle-income countries (LMICs)

**DOI:** 10.3389/fpubh.2023.1153971

**Published:** 2023-02-15

**Authors:** Ann Borda, Andreea Molnar, Michelle Heys, Christine Musyimi, Patty Kostkova

**Affiliations:** ^1^Faculty of Medicine, Dental and Health Sciences, University of Melbourne, Melbourne, VIC, Australia; ^2^Department of Information Studies, University College London, London, United Kingdom; ^3^Department of Computing Technologies, Swinburne University, Melbourne, VIC, Australia; ^4^UCL Great Ormond Street Institute of Child Health (GOS ICH), University College London, London, United Kingdom; ^5^Africa Mental Health Training and Research Foundation (AMHRTF), Nairobi, Kenya; ^6^UCL Centre for Digital Public Heath in Emergencies (dPHE), University College London, London, United Kingdom

**Keywords:** LMICs, digital interventions, serious mobile games, citizen participation, digital health technologies, mhealth, public health

In recent years, digital technology has demonstrated significant potential for delivering public health, health systems and health interventions remotely in low- and middle-income countries (LMICs) (1). Particularly, mobile health (mhealth) applications and serious mobile games (games with a “serious” aim, e.g., improving knowledge or changing behavior) (2) and open-source technologies have promising evidence in supporting improved efficacy of public health solutions in LMIC settings (3). These digital interventions span across maternal and neonatal health, mental health, communicable and non-communicable diseases, clinical decision support, surveillance, and health communication.

There are existing challenges, however, to address for the effective development, design and implementation of these interventions, such as standard frameworks (4), equity and ethics (5), and sustainability in citizen health participation and practice in LMIC regions (6). For example, citizen participation is often digitally-based, excluding those groups without access to the technology (7). There is a resulting need to consider a range of participatory and narrative methods within this context, accounting for lived experience and local traditional knowledge and other means of empowerment (8), including innovations in participation for diverse communities (9).

A critical underpinning to impactful participation and adoption is digital literacy. Meherali et al. conducted a systematic review of digital literacy aimed to empower adolescent girls from LMICs–mainly those in Africa, Asia and South America. The authors found that digital literacy interventions, such as mhealth and internet-based tools, can support adolescent girls to access certain resources across health information and education. However, with other services, such as financial and economic, the evidence is inconclusive. The authors recommend that long-term studies are needed in this area to better determine the impact of these interventions.

A complementary literature review by Ouedraogo et al. provides a perspective on serious mobile game use and health literacy in Sub-saharan Africa, especially among youth and young adults who have increasing access to inexpensive phones. The review focuses on the availability and implementation of serious mobile games aimed at enabling the promotion of healthy behavior change in rural communities. Findings suggest that mobile serious games could enhance access to health information and thereby contribute to healthy behavior change, but due to limited resources in rural areas, implementing sustainable mhealth interventions remain a challenge, including barriers like internet connectivity, language, and low literacy.

These considerations are developed further in the evaluation of mobile serious game application, MANTRA (Mueller et al.) aimed at vulnerable low-literacy women and girls in rural Nepal. Building on participatory approaches for public health (10), MANTRA was developed through a co-creation development process with local stakeholders and rural women (11). During the 2015 Nepal earthquake, pregnant and perinatal women faced major challenges and disruptions to their healthcare. In this Research Topic, Mueller et al. assessed the impact of MANTRA on maternal and child health resilience and awareness of geohazards. MANTRA teaches 28 learning objectives and danger signs in geohazards, maternal, and neonatal health to improve knowledge and self-assessment of common conditions and risks to inform healthcare-seeking behavior. The evaluation results reveal significant knowledge gain in participants playing the serious game on mobile phones, suggestive of promising educational impacts, and opportunity for scaling up the work.

Kayastha et al. extended the MANTRA study to explore the perceptions and usability of the serious game app amongst rural Nepalese women and Female Community Health Volunteers providing basic primary health support and education in rural settings. Despite the challenges of a user group with limited educational levels and low smartphone experience, participants were willing to engage further with the mhealth intervention and to share their experience and knowledge with fellow community members. The MANTRA pilot highlights women's positive perceptions of mhealth, and contextualizes participant's knowledge gain and informed decision-making in maternal and neonatal health problems.

Almost two-thirds of the 2.4 million newborn deaths worldwide annually could be avoided through equitable implementation of existing low-cost evidence-based interventions (12). This could be addressed by supporting healthcare providers to deliver standardized high-quality newborn care in clinical settings where 90% of the world's mothers now deliver. Crehan et al. give insights into a real-world usability pilot of a mhealth application conducted in a hospital neonatal unit in Malawi. The Neotree is an open-source digital quality improvement system co-created with newborn healthcare providers and caregivers in Malawi, Zimbabwe and Bangladesh (3, 13). It combines immediate data capture, education, and clinical decision support in a mhealth app for healthcare professionals working in low-resource neonatal units. The present study describes the usability-focused optimisation of the Neotree app, combining agile methods with thematic analysis and iterative user refinement. System usability of the Neotree app remained high after refinements and usage of the app by 93 neonatal nurses as an intervention for 1,323 babies. The study demonstrated that optimisation of a mhealth app in a low-resource setting could potentially inform usability studies of apps in similar settings.

Other public health concerns such as mosquito-borne diseases place a huge public health burden on the inhabitants of tropical regions in LMICs, and surveillance is a crucial process for understanding the population dynamics of mosquitoes (14, 15) as well as predicting local weather and climatic conditions (16). Scaling a digital public health intervention for mosquito surveillance is the focus of the paper by Aldosery et al.. The authors describe the process of co-designing and implementing a mosquito surveillance system (MEWAR) in two Brazilian cities with significant input by environmental health surveillance agents. The study outcome for the MEWAR pilot suggests the potential transferability of learnings to other intervention tools and for possible adoption by other LMICs.

This collection of research papers underscores that digital health interventions, such as serious mobile games and open-source mhealth applications, are opportunities to overcome various healthcare challenges in LMICs, by:

- Addressing limited access to health information due to social barriers like low-literacy and gender disparity.
- Developing digital interventions which collect and provide information about ways of responding to public health problems and which can supplement rural and regional health infrastructures.
- Strengthening health systems and delivery of universal healthcare through provision of open-source data-driven solutions for improving clinical care at the point-of-care in low-resource healthcare settings.

Reflecting on policy and practice implications, the effective adoption of digital interventions in LMICs is intrinsically founded on an understanding of social, economic, and cultural contexts in which the intervention will be implemented. Successful adoption can further address a range of Sustainable Development Goals, such as SDG3: Good health and wellbeing, SDG5: Gender equality, and SDG11: Reduced inequality.

Critically, this Research Topic highlights key factors in achieving resilient and sustainable digital health interventions in LMICs, including: participation of community members and experts by experience in co-development and/or deployment of the intervention, ability to easily share knowledge, low-cost or free to the user, and sufficient funding and resource support (e.g., health workforce capacity) to integrate the solution into a public health routine.

## Author contributions

AB wrote the editorial with all authors equally providing comments and approving the submitted version.

## References

[1] World Health Organisation. WHO Guideline: Recommendations on Digital Interventions for Health System Strengthening. Geneva: World Health Organization (2019). Available online at: https://www.who.int/publications/i/item/9789241550505 (accessed January 10, 2023). 31162915

[2] Wang Y Wang Z Liu G Wang Z Wang Q Yan Y. Application of serious games in health care: scoping review and bibliometric analysis. Front Public Health. (2022) 10:896974. 10.3389/fpubh.2022.896974 35757619 PMC9231462

[3] Heys M Kesler E Sassoon Y Wilson E Fitzgerald F Gannon H. Development and implementation experience of a learning healthcare system for facility based newborn care in low resource settings: the Neotree. Learn Health Syst. (2022) 7:e10310. 10.1002/lrh2.10310 36654803 PMC9835040

[4] Hoogendoorn P Versluis A van Kampen S McCay C Leahy M Bijlsma M. What makes a quality health app-developing a global research-based health app quality assessment framework for CEN-ISO/TS 82304-2: delphi study. JMIR Form Res. (2023) 7:e43905. 10.2196/43905 36538379 PMC9872976

[5] Shaw JA Donia J. The sociotechnical ethics of digital health: a critique and extension of approaches from bioethics. Front Digit Health. (2021) 3:725088. 10.3389/fdgth.2021.725088 34713196 PMC8521799

[6] Iyamu I Gómez-Ramírez O Xu AX Chang HJ Watt S Mckee G. Challenges in the development of digital public health interventions and mapped solutions: findings from a scoping review. Digit Health. (2022) 8:20552076221102255. 10.1177/20552076221102255 35656283 PMC9152201

[7] Sieck CJ Sheon A Ancker JS Castek J Callahan B Siefer A. Digital inclusion as a social determinant of health. NPJ Digit Med. (2021) 4:52. 10.1038/s41746-021-00413-8 33731887 PMC7969595

[8] Labrique AB Wadhwani C Williams KA Lamptey P Hesp C Luk R. Best practices in scaling digital health in low and middle income countries. Glob Health. (2018) 14:103. 10.1186/s12992-018-0424-z 30390686 PMC6215624

[9] Kyratsis Y Scarbrough H Begley A Denis JL. Editorial: digital health adoption: looking beyond the role of technology. Front Digit Health. (2022) 4:989003. 10.3389/fdgth.2022.989003 36419872 PMC9677232

[10] Borda A Molnar A Kostkova P. Serious games and participatory research in public health. In: DPH2019: Proceedings of the 9th International Conference on Digital Public Health. Marseille (2019). p. 133. 10.1145/3357729.3357762

[11] Mueller S Soriano D Boscor A Saville N Arjyal A Baral S. MANTRA: development and localization of a mobile educational health game targeting low literacy players in low and middle income countries. BMC Public Health. (2020) 20:1171. 10.1186/s12889-020-09246-8 32723317 PMC7385876

[12] Bhutta ZA Das JK Bahl R Lawn JE Salam RA Paul VK. Can available interventions end preventable deaths in mothers, newborn babies, and stillbirths, and at what cost? Lancet. (2014) 384:347–70. 10.1016/S0140-6736(14)60792-3 24853604

[13] Khan N Crehan C Hull-Bailey T Normand C Larsson L. Software development process of Neotree—a data capture and decision support system to improve newborn healthcare in low-resource settings. Wellcome Open Res. (2022) 7:305. 10.12688/wellcomeopenres.18423.1 PMC10682609 38022734

[14] Kostkova P Pinheiro dos Santos W Massoni T. ZIKA: improved surveillance and forecast of Zika virus in Brazil. Euro J Public Health. (2019) 29:414–5. 10.1093/eurpub/ckz186.085

[15] Musah A Rubio-Solis A Birjovanu G dos Santos WP Massoni T Kostkova P. Assessing the relationship between various climatic risk factors & mosquito abundance in Recife, Brazil. In: DPH2019: Proceedings of the 9th International Conference on Digital Public Health. Marseille (2019). p. 97–100. 10.1145/3357729.3357744

[16] Musah A Dutra LMM Aldosery A Browning E Ambrizzi T Borges IVG. An evaluation of the OpenWeatherMap API versus INMET using weather data from two Brazilian cities: Recife and Campina Grande. Data. (2022) 7:106. 10.3390/data7080106

